# Successful Control of Acute Myelofibrosis with Lenalidomide

**DOI:** 10.1155/2010/421239

**Published:** 2011-01-17

**Authors:** G. Vassilopoulos, M. Palassopoulou, K. Zisaki, M. Befani, E. Bouronikou, N. Giannakoulas, E. Stathopoulou, P. Matsouka

**Affiliations:** ^1^Division of Hematology, University Hospital of Larisa, 41110 Biopolis, Greece; ^2^Genetics and Gene Therapy Lab, Biomedical Research Foundation, Academy of Athens, Soranou tou Efesiou 4, 11527 Athens, Greece; ^3^Medical Department of Hematology, GENESIS Pharma SA, 274 Kifisias Avenue, Halandri, 15232 Athens, Greece

## Abstract

Acute panmyelosis with myelofibrosis (APMF) is a rare, fatal hematological neoplasm that is characterized by the acute onset of cytopenias and fibrosis in the bone marrow in the absence of splenomegaly or fibrosis-related morphological changes in the RBCs. We present the case of a 59-year-old female who presented with a two-month history of anemia, leucopenia and a normal platelet count. The marrow was heavily fibrotic, and no aspirate material could be obtained; the biopsy showed extensive infiltration with small to medium size megakaryocytes, dysplastic changes in the erythroid compartment, and left shift in the myeloid cells. The patient was treated for four months with anabolic steroids (Danazol), growth factors and received regular blood transfusions. At 4 months after diagnosis, the patient was started on Lenalidomide, 10 mg/day for a 21-d-course along with growth factor support. At 6 months after treatment, the patient was transfusion-independent, had normalized blood counts, and, at 32 months on continuous lenalidomide treatment, her needs for growth factor support have been minimized. Repeat bone marrow biopsies showed a patchy distribution of fibrosis with areas of normal cellularity and morphology. To our knowledge, this is the first case for a medication that could reverse the fatal outcome of APMF.

## 1. Introduction

Acute panmyelosis with myelofibrosis (APMF) is a rare disease entity that was initially described as “malignant myelosclerosis” [1] almost 50 years ago. In the recent WHO classification, the term acute panmyelosis with myelofibrosis (APMF, ICD-O code 9931/3) has been coined to describe an identical clinical syndrome characterized by constitutional symptoms, cytopenias, absence of splenomegaly and of related morphological changes in the RBCs; the disorder runs a rapid and usually fatal course. Histologically, APMF is characterized by a heavily fibrotic marrow, an increased cellularity with the presence of immature precursors from all three lineages (panmyelosis), and an abnormal population of megakaryocytes [2]. The latter show dysplastic features with under- or nonlobulated nuclei, dispersed chromatin, and a high proportion of small-sized cells. The condition should be differentiated from other hematological neoplasms that present with fibrosis such as acute megakaryoblastic leukemia and MDS with fibrosis; in the former, the presence of more than 20% blasts with at least half of them expressing megakaryocyte markers confirms the diagnosis, while, in the latter, the distinction is based on the subacute onset and the presence of dysplastic features characteristic of MDS. At the clinical level, APMF is characterized by rapid progression and a fatal outcome usually within months from diagnosis; in a relatively large series of 46 patients, 76% succumbed to severe bone marrow failure, and 22% developed acute myeloid leukemia [3]. 

One medication that has therapeutic potential in MDS with the 5q-karyotype and marginal efficacy in myelofibrosis is lenalidomide [4, 5]. A feature of the 5q-MDS is the increased number of megakaryocytes with no fibrosis in the bone marrow. The precise mechanism of lenalidomide's action is uncertain since it has pleiotropic effects and belongs to a new generation of drugs known as immunomodulatory (IMiDs). However, there is evidence that lenalidomide has antiproliferative potential on the MDS clone, can alter the T- and NK-cell responses, can downregulate cytokine expression, and has an effect on vascular proliferation by modulating VEGF expression [6]. A common side effect of lenalidomide treatment is reversible thrombocytopenia indicating that the formulation may have a specific, yet unidentified, action on megakaryocyte proliferation. Based on this hypothesis and on the confirmed action of lenalidomide in the 5q-MDS and in primary myelofibrosis, we sought to investigate its potential for the treatment of APMF.

## 2. Case Report

**Figure 1:**
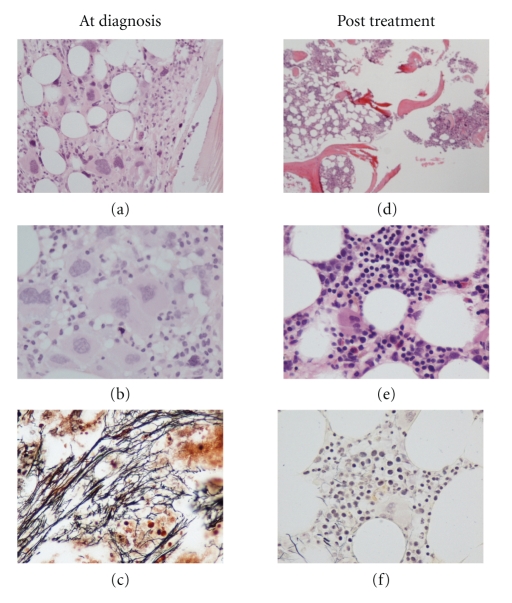
(a, b, c) relatively hypercellular marrow with marked fibrosis, abundant atypical megakaryocytes, and scattered immature-appearing cells, (a, b) H&E-stained sections, (c) reticulin stain). (d, e, f) following treatment with lenalidomide, the bone marrow showed patchy hematopoiesis with areas of high and low cellularity ((d) H&E-stained section), decreased numbers of megakaryocytes ((e) H&E-stained section) and a marked reduction in reticulin fibrosis ((f) reticulin stain).

A 59-year-old female was referred to our unit for the investigation of anemia; the patient complained of fatigue and malaise that started about one month prior to her visit. She did not mention any other constitutional symptoms such as fever or weight loss. Her past medical history revealed that she had mild mitral and aortic valve insufficiency along with atrial fibrillation and was on digitalis, an ACE inhibitor and acenocoumarol with a target INR of 2.5. On physical examination, she was pale, afebrile, had no palpable lymph nodes, and her abdomen did not disclose any organomegaly. Her CBC showed severe anemia with a hemoglobin level of 7.2 g/dL, an MCV of 94.8 fL, leucopenia (3400/uL, with a differential of 56% PMN, 38% lymphocytes, 5% mononuclears, and 1% eosinophils). The platelet count was elevated (756,000/uL), and some giant forms could be observed in the smear. Microscopic examination of the peripheral blood did not show any abnormalities associated with myelofibrosis, while the WBC had no dysplastic features. Serum parameters were normal except for ferritin (12 × the upper limit of normal, ULN) and LDH (1.8 × the ULN). Bone marrow aspiration did not yield any material, and it was considered as “dry tap.” Pathology revealed increased cellularity and fibrosis; there was prominent hyperplasia of the megakaryocytic lineage with clustering and dysplastic features (Figures 1(a) to 1(c)). Few (less than 5%) small sized CD61 positive cells with prominent nucleoli were also observed which however did not express CD34. Hyperplasia was also noted in the myeloid lineage with a left shift and a normal blast count; the erythroid lineage was proportionally diminished. Molecular analysis for *bcr-abl* and JAK2V617F mutation were negative. Based on the available findings, the acute onset, and the absence of splenomegaly, the diagnosis of primary myelofibrosis was excluded; the picture was consistent with either a case of acute myelofibrosi or a secondary fibrosis accompanying either an atypical myeloproliferative disorder or an MDS. The possibility of reactive fibrosis secondary to an infiltration of malignant cells was excluded based on negative clinical, radiological, and histochemical findings. 

The patient was transfused regularly and was started on ESAs (epoetin alfa, 40.000 iu per week) and oral danazol (200 mg qd). At two months following her admission, she was still transfusion-dependent, and her blood counts were deteriorating, her hemoglobin level was at 6.5 gr/dL, her WBCs were at 3,400/uL with normal differential and the platelets were at 224,000/uL. A repeat bone marrow was performed at 5 months after presentation with an identical picture on the biopsy; the marrow was still a “dry tap,” but, of the few progenitors, a karyotype was obtained that showed a normal 46; XX chromosomal pattern. A marginal increase in the myeloid blasts was also noted, but the overall number of CD34+/cKit+ cells was less than 3%.

At this point, a decision was made to withhold danazol and start on lenalidomide at a dose of 10 mg qd for 21 days of 28 days cycles, while growth factor support was maintained. The patient tolerated the therapy well, and, within 3 months, her transfusion requirements subsided, and she experienced a gradual increase in her hemoglobin levels that reached 15 gr/dL at the 10th monthly lenalidomide cycle. At this point, her WBC count and differential were normal, and she had consistently lower than normal PLT levels (135,000/uL on average); growth factor support with ESA and G-CSF has continued, but the needs have been cut to half. A repeat bone marrow was performed at the 18th cycle of lenalidomide which for the first time showed a patchy distribution of cellularity; however, in areas of high cellularity, an identical clustering of the abnormal megakaryocytes could be observed (Figures 1(c) to 1(d)). Of the other lineages, the erythroid showed dysplastic features, while, in the myeloid compartment, a marginally elevated number of immature cells was recorded (4%-5% blasts of myeloid lineage). 

Currently, at almost three years after her initial admission, the patient has stabilized her counts requiring ESA every 21 days and GCSF every other week. No morphological changes can be observed in her peripheral blood, and no splenomegaly has developed.

## 3. Discussion

Acute myelofibrosis refers to a number of disorders that present with cytopenias, absence of splenomegaly, and a clinical course of fatal peripheral blood insufficiencies or the development of leukemias. A number of disease entities could present with such a picture such as acute panmyelosis with myelofibrosis (APMF), MDS with fibrosis, AML of the M7 subtype, and unspecified myeloproliferative neoplasms with fibrosis. Our patient filled the criteria of APMF as specified in the new WHO listing of hematological neoplasms [2]; the patient had a typical clinical course, had no morphological abnormalities in the peripheral blood, and no splenomegaly, no megakaryoblasts but an abundant infiltration of abnormal megakaryocytes (MGK) and reticulin deposition in the bone marrow. According to what has been previously published [3], a prominent feature of APMF that was also present in our patient is the presence of MGK clusters (32% of cases) and microMGK with maturation defects (96% of cases). Of the other disease features, the sole unique abnormality in our patient was an elevated platelet count at presentation that later fell below normal levels and normalized after the 3rd course of lenalidomide. In addition, we could not confirm any other cause of reactive fibrosis since neither digitalis, ACE inhibitors, nor acenocoumarol has been linked to myelofibrosis. The possibility that our patient was an atypical case of a 5q-MDS cannot be supported based on a normal karyotype, lack of dysplastic features in the peripheral, and presence of reticulin fibrosis. However, APMF cases with partial loss of 5q have been observed in one series [7], while aberrations of chromosome 7 were recorded in a significant number of cases usually as part of a complex karyotype. The distinction to the classical primary myelofibrosis (PMF) is relatively straightforward; a brief outline of the major features that characterize APMF, PMF, and the 5q-syndrome are presented in Table 1.

The bone marrow in the published APMF series is consistently inaspirable; in cases where metaphases have been obtained [7], a partial loss of 5q has been described, either as the sole abnormality or as along with other abnormalities such as monosomy 7 and trisomy 8. These chromosomal aberrations are consistent with an aggressive myeloid neoplasm which however culminates in AML only in a minority of cases. In our patient, we could only analyze one sample that was obtained after 15 months on lenalidomide; of the 7 metaphases, all had a normal female karyotype. Although normal karyotypes in APMF have been described, the possibility remains that the abnormal clone may be extinct due to lenalidomide treatment.

APMF is a rare disease entity accounting for less than 1% of all hematological neoplasms and has a fatal course usually within months from diagnosis. In a series of 46 patients [3], 76% of patients succumbed to hemorrhage or infections, and 22% developed AML, while the median survival was 9 months. This dismal prognosis has been reverted in our patient with the administration of lenalidomide that had an impact on both the clinical picture and the BM fibrosis. The exact action of lenalidomide is still unknown, but it has immunomodulatory, antiangiogenic, and direct antitumor effects mediated by an increase in Th1 cytokines and NK cell activity [6]. In the case of APMF, the pathogenesis is obscure, but alterations in the BM vasculature, the cytokine network, and the immunological milieu have all been described [3]. The documented efficiency of lenalidomide in the treatment of the 5q-MDS and in our case study cannot be attributed to any specific component of its pleiotropic actions.

The pathogenesis of APMF is unknown, and the rarity of the disease makes its study a medical challenge. Mutations in the JAK-STAT kinase pathway or the MPL receptor have not been recorded, and there is no specific cytogenetic abnormality; however, there is a predilection for complex karyotypes involving both chromosomes 5 and 7, similar to MDS and acute leukemias. In the series of 46 patients, exposure to a toxic agent was identified in 3 patients [3], while in our case we could not identify any offending agent; our patient was not occupied and would only occasionally help her husband's farming activities. A recurring feature in all patients with APMF is the presence of abnormalities particularly in the megakaryocytes, which have been implicated in the development of idiopathic myelofibrosis [8]. In the latter, MKs are produced in higher rates by the abnormal HSC clone, and they produce large amounts of TGFb, a cytokine that plays a crucial role in the development of marrow fibrosis [9]. In addition, TGFb upregulates osteoprotegerin expression which in turn inhibits osteoclasts and results in the increased deposition of extracellular matrix in effect altering the bone marrow microenvironment. 

There are no consensus standards for the treatment of APMF. A variety of therapeutic strategies have been employed without success. Nontoxic agents such as zoledronic acid and danazol have produced remissions, while a single case of autologous stem cell transplantation has been reported [10] that resulted in 9 years of remission. Thalidomide and its analog lenalidomide are antiangiogenic and immunomodulatory agents that inhibit several proinflammatory cytokines. They also have direct antitumor activity in myeloma and myelodysplastic cell lines. Their use in patient with idiopathic myelofibrosis has been marginally successful [5], while lenalidomide has been approved for the MDS 5q-disorder. We used lenalidomide in our patient on the basis of its beneficial effect in patients with idiopathic myelofibrosis and the 5q-syndrome. Currently, at almost 3 years on continuous lenalidomide treatment, we started an alternate month administration scheme and have further reduced growth factor support. Although lenalidomide's action in APMF has not been reported before, if replicated in other cases, it could provide a treatment option for a lethal disorder.

## Figures and Tables

**Table 1:** The basic characteristics that help in the differential diagnosis of APMF (acute panmyelosis with myelofibrosis), PMF (primary myelofibrosis), and the 5q-syndrome according to the recent WHO classification (2008).

	APMF	PMF	5q-
MGK increase	Prominent	Present	Present
JAK2V617F	No	Yes (50%)	No
Acute onset	Yes	No	No
Splenomegaly	No	Yes	No
Marrow Fibrosis	Yes	Yes	No
Pancytopenia	Yes	No	No
